# Fetal echocardiography changes of the right ventricle of well-controlled gestational diabetes mellitus

**DOI:** 10.1186/s12872-023-03539-7

**Published:** 2023-10-06

**Authors:** Ying Ma, XueSong Sun, XiaoZhi Liu, LiHua Hu, Ye Song, Xiong Ye

**Affiliations:** 1 Department of Ultrasound, Zhou Pu Hospital, Shanghai University of Medicine & Health Sciences, Shanghai, China; 2 Department of Obstetrics and Gynecology, Zhou Pu Hospital, Shanghai University of Medicine & Health Sciences, Shanghai, China; 3 School of Clinical Medicine, Shanghai University of Medicine & Health Sciences, Shanghai, China

**Keywords:** Fetal echocardiography, Gestational diabetes mellitus, Right ventricle, Speckle tracking, Strain

## Abstract

**Background:**

There is few evidence of right ventricular (RV) function in fetuses with gestational diabetes mellitus (GDM). Therefore, the aim of this study was to assess the RV function of fetuses using routine and two-dimensional speckle-tracking echocardiography (2D STE) to determine the effects of well-controlled GDM in the third trimester.

**Methods:**

We used a Philips Epiq7C ultrasound instrument to obtain RV data sets from 63 subjects from July 2019 to February 2022. We compared the free wall thickness (FWT), fractional area change (FAC), Tei index (TEI), tricuspid annular plane systolic excursion (TAPSE) and free wall longitudinal strain(FWLS)of the RV in mothers with well-controlled GDM and normal gestational age-matched fetuses.

**Results:**

63 third trimester fetuses (32 GDM; 31 healthy controls) met the enrolment criteria. Significant differences in fetal RV were detected between the GDM and control groups for the FAC (36.35 ± 6.19 vs. 41.59 ± 9.11; P = 0.008) and the FWLS (-18.28 ± 4.23 vs. -20.98 ± 5.49; P = 0.021). There was a significant difference among the segmental strains of the base, middle and apex of the RV free wall in the healthy controls (P = 0.003), but in the GDM group, there was no statistical difference (p = 0.076). RV FWLS had a strong correlation with FAC (r = 0.467; P = 0.0002).

**Conclusions:**

In well-controlled GDM, there was measurable fetal RV hypertrophy and significant systolic function decline, indicating the presence of ventricular remodeling and dysfunction. 2D-STE can evaluate the RV free wall contraction in a more comprehensive way.

## Introduction

Gestational diabetes mellitus (GDM) is defined as any degree of glucose intolerance, onset or first detected during pregnancy [1], with a prevalence of 9–25% of all pregnancies worldwide in the last decade [2]. The prevalence of GDM in China is approximately 14.8% [3]. GDM is associated with adverse neonatal outcomes, including the risk of developing myocardial dysfunction and neonatal morbidity, even in good glycemic control [4]. Despite good glycemic control, 30% of diabetic mothers can cause fetal hypertrophic cardiomyopathy, sometimes leading to sudden intrauterine fetal death [5, 6]. Echocardiography is a noninvasive screening tool for the assessment of cardiac structure and function of fetuses. The review of Depla’s showed that presentational or gestational maternal diabetes is associated with fetal cardiac hypertrophy, diastolic dysfunction and impaired global myocardial function on obstetric ultrasonography [4].

There is little evidence currently available to estimate right myocardial damage that especially occurs in GDM. The fetal central circulation is a very flexible and adaptable circulatory system which differs from adults [7, 8]. At 28 to 32 weeks, the venous duct and foramen ovale shunt flow reached the minimum, and the partial lung flow reached the maximum [9]. The right heart occupies the advantage position of fetal circulation, right cardiac output accounts for 60 to 70% of total output, and the RV forms a greater proportion of the total ventricular weight than it does in childhood or adulthood [10]. Under these physiological baselines, the hemodynamic properties and functional range of these shunts are important determinants of fetal cardiac and circulatory development in the second and third trimesters [11].

Two-dimensional speckle tracking echocardiography (2D STE) is an imaging algorithm for changes in cardiac function [12, 13]. 2D STE has no dependence on ultrasound beam angulation and relatively small operator dependence [13]. Strain parameters are better than ejection fraction and have allowed detection of preclinical myocardial dysfunction [14]. It was found that the change in fetal myocardial strain occurred earlier than the traditional ultrasonic estimate of cardiac function [15, 16].

In this study, we aimed to explore the echocardiography changes of systolic function of fetal RV using traditional and 2D STE in well-controlled GDM mothers.

## Materials and methods

### Subjects

This prospective observational cross-sectional study included 32 fetuses (35.78 ± 0.790 weeks) of mothers with GDM as the case group and another 31 gestational age-matched fetuses (36.42 weeks ± 0.773) of normal pregnancies as the control group. Cases and controls were recruited from July 2019 to February 2022 at the Department of Ultrasound, Zhou Pu Hospital Affiliated to Shanghai University of Medicine & Health Sciences. Maternal medical records were reviewed in all subjects, including body mass index (BMI) and blood pressure.

### Inclusion and exclusion criteria

**Inclusion criteria**: The cases were as follows: mothers aged 18 years or older with GDM as per the International Association of Diabetes and Pregnancy Study Group criteria [1] in the third trimester at Zhou Pu Hospital. The control group was normal pregnancies matched by gestational age. All the mothers with GDM only through diet to controlled their blood glucose (fasting blood glucose < 5.3mmol/L), and did not use oral hypoglycemic drugs or injected insulin.

**The exclusion criteria were as follows:** (1) fetal structural heart disease; (2) twin or multiple pregnancies; (3) pregnant women with hypertensive syndrome during pregnancy, hyperhydramnios, chronic kidney disease, liver disease, rheumatic disease, thyroid disease, autoimmune disease, etc.; (4) The anterior chest wall of the fetus does not face to or the placenta is located in the anterior wall of the uterus, and the distance of the fetus is far from the abdominal wall of the mother. (The fetal heart structure will cannot be clearly showed, and it is difficult to obtain a standard section to fit for measuring the cardiac function of the fetus.)

### Equipment and ultrasonic image acquisition

#### Equipment

A Philips Epiq7C color Doppler ultrasound diagnostic instrument (Philips Healthcare, Andover, MA) was adopted with c5-1 (5–10 MHz) probes. It was equipped with an offline Q-Lab 13.0 ultrasonic image workstation that has special software for right ventricular automatic strain quantitative analysis.

**Ultrasonic image acquisition**: Echocardiograms were performed at a single clinical site. All ultrasonic operations followed published guidelines for fetal echocardiography [17]. Image acquisition adjustments, such as sector width, depth, gain, focus or function of frequency and tissue harmonic, were used to optimize the frame rate (> 80 frames/Sect. ) [18]. A dynamic image dataset of 2 to 4 s was captured in the following views: 4-chamber view of the apical or basal fetal cross-section. Two experienced echo cardiographers (Ying Ma and LiHua Hu) performed the fetal echocardiography. All parameters were measured 3 times, and the average value was taken for analysis. Patients’ diagnosis was blinded to operators at the time of the ultrasound image acquisition and offline analysis.

### Fetal echocardiography

#### Conventional two-dimensional doppler fetal echocardiography (Fig. 1)

**Fig. 1:**
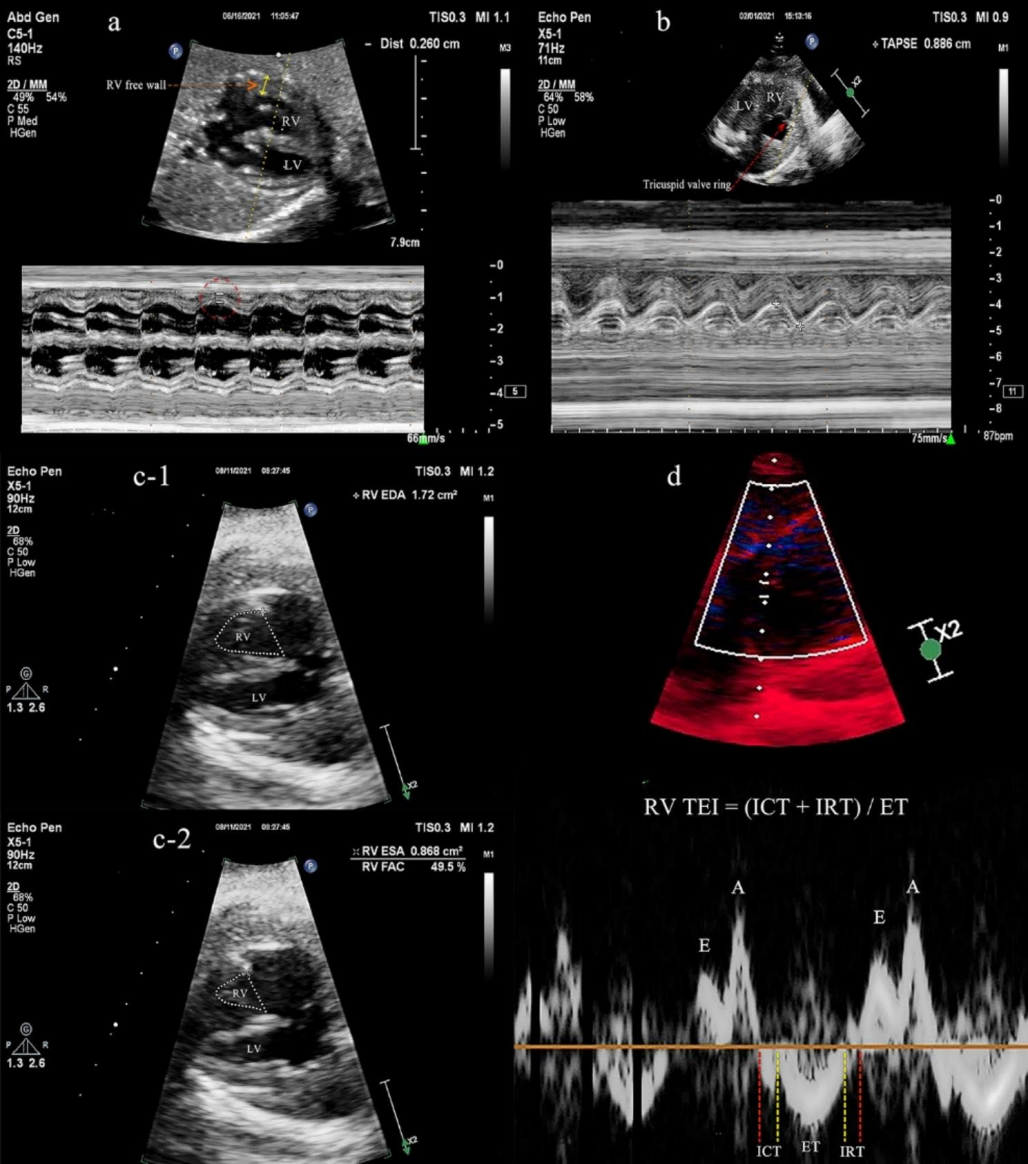
Conventional fetal echocardiography (**a**) Right ventricular free wall thickness; (**b**) Tricuspid annular plane systolic excursion, longitudinal displacement of the tricuspid annulus from end systolic to end diastolic measured on the obtained tricuspid annulus motion curve; c-1-2. Right ventricular fractional area changes, (end-diastolic area - end-systolic area)/end-diastolic area ×100%; d. Right ventricular Tei index (RV TEI = (ICT + IRT)/ET, *ICT, isovolumetric contraction time; IRT, isovolumetric relaxation time;* ET, ejection time)

1) Right ventricular free wall thickness (RV FWT): M-mode was obtained perpendicular to the RV long axis in a 4-chambered view of the fetal heart to assess the RV FWT (Fig. 1a);

2) Tricuspid annular plane systolic excursion (TAPSE): The motion spectrum of the tricuspid annulus was measured by M-mode. According to the size of the heart, let the sampling line be consistent with the movement direction of the long axis of the RV at an angle of < 20°, place the sampling line at the junction of the anterior tricuspid valve ring and the RV free wall, and obtain the motion spectrum of the tricuspid valve ring. TAPSE is the longitudinal displacement of the tricuspid annulus from end systolic to end diastolic measured on the obtained tricuspid annulus motion curve (Fig. 1b);

3) Right ventricular fractional area changes (RV FAC): The edges of the RV at the end diastolic and end systolic endocardium were recorded. RV FAC = (end-diastolic area - end-systolic area)/end-diastolic area ×100% (Fig. 1c);

4) Right ventricular Tei index (RV TEI): In the fetal 4-chamber view, the tissue Doppler sampling gate was placed on the lateral ring of the tricuspid valve to obtain the Doppler spectrum and calculate the RV TEI index. The RV TEI index is calculated by measuring time intervals including isovolumic contraction time (ICT) from tricuspid valve closure to pulmonic valve opening; isovolumic relaxation time (IRT) from pulmonic valve closing to tricuspid valve opening; and ejection time (ET) from pulmonic valve opening to closing. RV TEI = (ICT + IRT)/ET, as described previously [19] (Fig. 1d).

#### Two-dimensional ultrasound speckle tracking technique

**Fig. 2:**
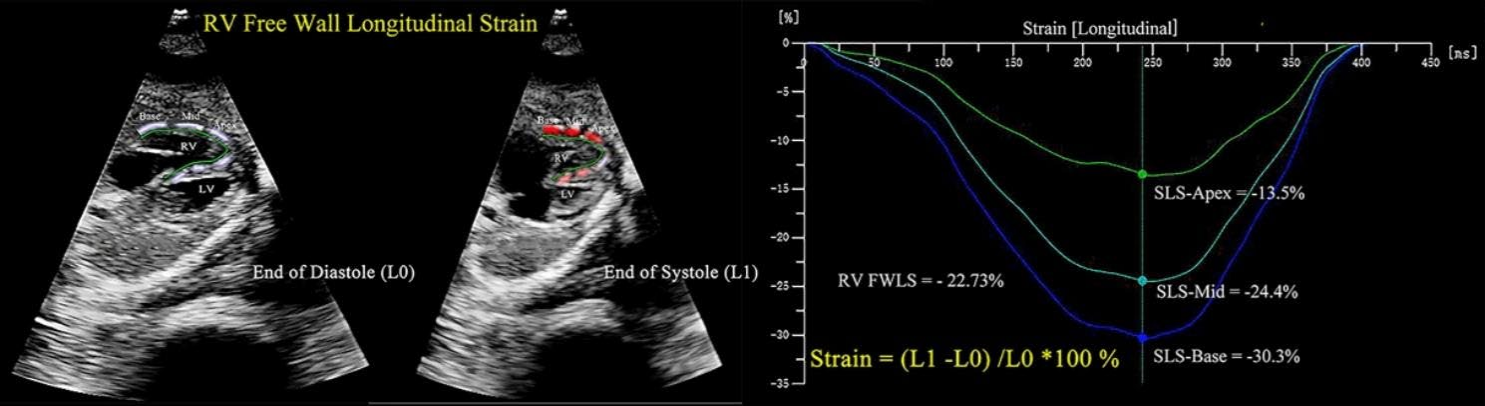
Offline analysis of fetal right ventricular free wall segmental and integral longitudinal strains using Q-Lab 13.0. The initial length (L0) of the end diastolic of the three segments is shown on the left, and the length of the end systole (L1) is shown on the right

RV ultrasound images of fetal 4-chamber hearts were collected as two-dimensional gray scale images within three to four consecutive cardiac cycles. All images were analyzed by Q-Lab offline software using DICOM data without any loss of frame rate. From the 4-chamber apical view, the software automatically delineated the endocardial boundary of the RV and manually fine-tuned the systolic and diastolic boundaries to track the whole RV. The analysis software automatically divided the free wall of the RV into three equal-sized sections (basal, middle, and apical), tracking longitudinal strain in the region of interest (ROI). Longitudinal strain (LS) of the basal, middle and apical segments of the RV free wall is defined as the relative shortening of an ROI between the entire endocardial contour length at the end of diastole (L0) and end of systole (L1) (LS = (L1 – L0)/L0 × 100%) and by convention is expressed as a negative percentage [20]. The peak systolic RV free wall longitudinal strain (FWLS) was calculated as the average of the basal, mid, and apical RV free walls (Fig. 2).

### Data analysis

We used SPSS version 25 (SPSS Inc., 2016, Armonk, NY) for all analyses. All variables were tested for normality before data were presented as either the mean ± standard deviation (SD) or error of mean (SE), as appropriate. Differences between the two groups were compared using unpaired t-tests, and three groups were subjected to analysis of variance (one-way ANOVA) according to data characteristics. Correlation was tested by Pearson’s correlation coefficient. P value < 0.05 was considered dominant.

## Results

### Characteristics of participants

Background characteristics are shown in Table 1. A total of 63 subjects were included for final analyses, and 31 maternal controls were aged 27.11 ± 4.15 years and 32 GDM cases aged 28.45 ± 3.78 years (p = 0.320). The mean ± SD of fasting blood glucose was 6.37 ± 1.61 mmol/L and HbA1c was 5.60% (range, 5.40–5.80%) in the GDM group when they be diagnosed. There was no significant difference in mother’s BMI, gestational age or fetal heart rate between the groups.

**Table 1:** Basic characteristic of pregnant women and fetuses (mean ± SD)

Variables	Control group (N = 31)		GDM group (N = 32)	P values		
**Maternal characteristics**						
*Maternal age (years)*	27.11 ± 4.15	28.45 ± 3.78		0.320		
*FBG(mmol/L)*	4.78 ± 0.78	6.37 ± 1.61		< 0.0001		
*HbA1c(%)*		5.60 ± 0.20				
*BMI (cm/s)*	26.28 ± 2.50	27.53 ± 4.62		0.073		
*Systolic pressure (mmHg)*	113.80 ± 1.77	122.40 ± 1.98		0.003		
*Diastolic pressure (mmHg)*	63.89 ± 1.24	68.11 ± 1.76		0.060		
**Fetal characteristics**						
*Gestational age (wk.)*	35.78 ± 0.79	36.42 ± 0.77			0.327	
*Fetal heart rate (beats/min)*	146.60 ± 9.37	146.00 ± 8.51			0.776	

BMI: body mass index; FBG: fasting blood glucose; GDM: gestational diabetes mellitus; HbA1c, glycated hemoglobin

### Parameters of fetal echocardiography

**Fig. 3:**
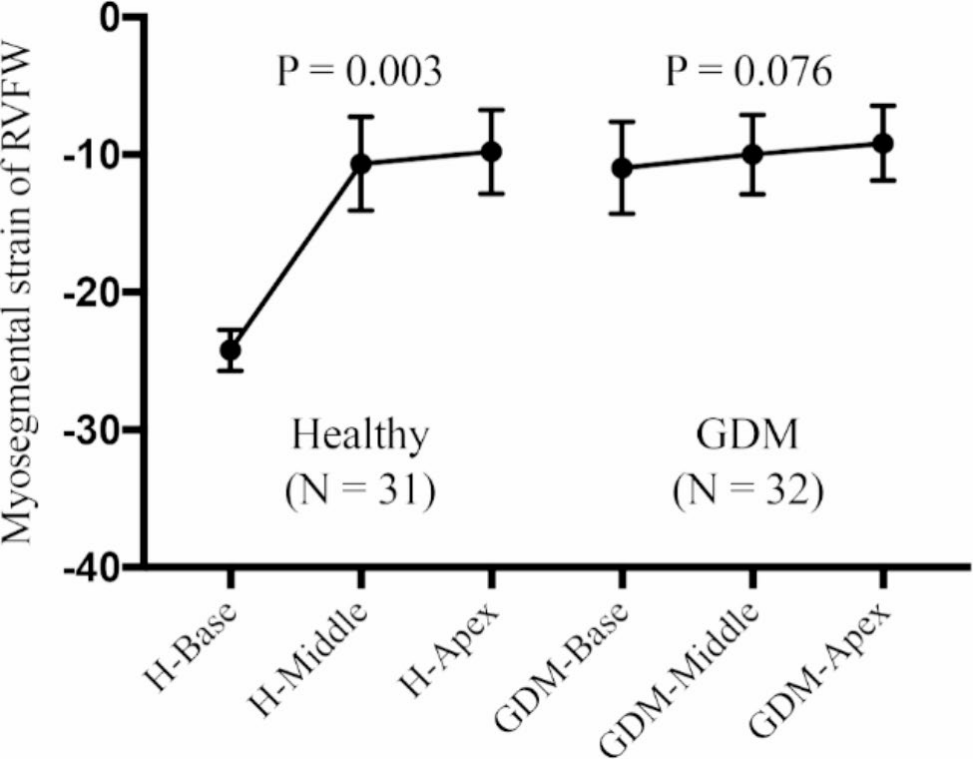
Comparison of strains in each myocardial segment of the fetal right ventricular free wall in healthy pregnant women and GDM mothers

The results of the 2D conventional fetal heart ultrasound examination and 2D-STE analyses are provided in Table 2. Fetal RV FWT, TEI and TAPSE were not different, but the RV FAC and FWLS were significantly less in the GDM group than in the control group (P = 0.008; P = 0.021, respectively). There was a significant difference among the segmental strains of the base, middle and apex of the RV free wall in the healthy control group, but in the GDM group, there was no statistically significant difference (Fig. 3).

**Table 2:** Fetal right ventricular changes in the control and GDM groups (mean ± SE)

Variables	Control group (n = 31)	GDM group (n = 32)	P values
*FWT(mm)*	2.41 ± 0.13	2.54 ± 0.09	0.460
*TEI*	0.45 ± 0.02	0.50 ± 0.02	0.054
*FAC(%)*	41.59 ± 1.82	36.35 ± 1.09	0.008
*TAPSE(mm)*	7.98 ± 1.07	7.84 ± 1.53	0.684
*SLS-Base*	-24.23 ± 8.28	-10.98 ± 18.90	0.001
*SLS-Mid*	-10.67 ± 1.49	-9.99 ± 3.34	0.306
*SLS-Apex*	-9.79 ± 3.42	-9.18 ± 2.71	0.380
*FWLS*	-20.98 ± 1.03	-18.28 ± 0.75	0.021

FWT: free wall thickness; FAC: fractional area change; FWLS: free wall longitudinal strain; GDM: gestational diabetes mellitus; TAPSE: tricuspid annular plane systolic excursion; TEI: Tei index; SLS-Apex: segmental longitudinal strain of apical; SLS-Base: segmental longitudinal strain of base; SLS-Mid: segmental longitudinal strain of middle

As shown in Table 3, when including all subjects (both healthy control and GDM groups), according to Pearson correlation analysis, we found that RV FWLS had a strong correlation with FAC (r = 0.467; P = 0.0002); gestational age was not correlated with RV TEI, FWT, TAPSE, FAC or FWLS.

**Table 3:** Correlation coefficients among study variables of fetal right ventricular echocardiography (n = 63, r values)

Items	FWLS	FAC	TEI	FWT	TAPSE	Gestational age
***FWLS***	1					
***FAC***	0.467#	1				
***TEI***	-0.222	-0.163	1			
***FWT***	-0.258	0.008	-0.363*	1		
***TAPSE***	-0.010	0.045	-0.097	0.003	1	
***Gestational age***	-0.200	-0.110	0.032	-0.114	0.052	1

FAC fractional area change, FWT free wall thickness, TEI: Tei index, TAPSE tricuspid annular plane systolic excursion; FWLS: free wall longitudinal strain. #P = 0.0002; *p = 0.007

### Intra-observer and inter-observer reproducibility

The reproducibility of RV overall and segmental free wall longitudinal strains was assessed in a subgroup of 15 randomly chosen subjects. Interclass correlation coefficients were moderate for both intra-observer and inter-observer analyses (Table 4).

**Table 4:** Intra- and inter-observer agreements of parameters of fetal right myocardial deformation on 2D STE (95% CI)

Parameters	Intra-observer	Inter-observer
*FWLS*	0.81 (0.65–0.82)	0.95 (0.87–0.98)
*SLS-Base*	0.87 (0.76–0.95)	0.82(0.77–0.95)
*SLS-Mid*	0.82 (0.75–0.92)	0.88 (0.75–0.92)
*SLS-Apex*	0.81 (0.64–0.89)	0.87 (0.69–0.96)

FWLS: free wall longitudinal strain; SLS-Base: segmental longitudinal strain of basal. SLS-Mid: segmental longitudinal strain of middle; SLS-Apex: segmental longitudinal strain of apical

## Discussion

Pregnancies affected by diabetes often result in abnormal fetal development, including altered growth and nutrient distribution as well as congenital malformations [21]. Mothers with GDM have increased insulin resistance that can lead to maternal hyperglycemia and increased glucose transport across the placenta, with resultant fetal hyperinsulinaemia, which increases the synthesis and deposition of fat and glycogen in myocardial cells [22]. Elevated HbA (1c) values during the 1st trimester were associated with fetal cardiovascular defects in offspring [23]. The pathophysiology of the effects of maternal diabetes on the fetal heart is multifactorial and not fully understood, including the hyperglycemic environment, activation of a series of cellular events, and changes in gene expression [24–26].

There are several interesting results in our study. First, compared with normal pregnant fetuses matched with gestational age, the RV FAC and FWLS decreased significantly (P = 0.008; P = 0.021). This indicates RV hypertrophy and dysfunction in the fetal heart of GDM fetuses. This predicted a decrease in myocardial contractility and elasticity in GDM fetuses. Fetal hyperinsulinemia and insulin-like growth factor-I promote cardiomyocyte hypertrophy, leading to decreased myocardial compliance and function [27–29]. Second, there were no significant differences in the longitudinal strains of the basal, middle and apical parts of the free wall of right ventricle in GDM fetuses (p = 0.076), but the differences were significant in healthy fetuses in different segments (p = 0.003). This result similar to other studies [30] suggest that GDM fetal myocardium has significant diffuse impairment of RV function. To our knowledge, no other study has demonstrated the presence or absence of abnormal echocardiographic patterns to define the segmental nature of RV dysfunction that accompanies GDM. The RV myocardium mainly consists of deep longitudinal fibres [31], which play a key role in global RV contracts. Peak global longitudinal strain remains the most reliable quantitative tool for assessing RV function in children [32].

Third, we found that RV FWLS had a strong positive correlation with the right heart remodeling parameter FAC if we included all the control and GDM groups. In our and Willruth A. M. et al.’s studies (150 healthy fetuses at between 13 and 39 weeks gestation) [30], the correlation between RV FWLS and gestational age did not reach significance. However, Giovanni DS’ study showed that there was significant correlation between gestational age and average peak longitudinal strain (r = − 0.73; P < 0.001) in 100 20–32-week-old normal fetuses [33]. Possible reasons for this heterogeneity may include the use of different ultrasound systems and speckle-tracking algorithms [34]. Our study adds to the evidence for altered fetal RV structure and contractions in GDM.

There are few limitations of our study. First, it is a single-center, cross-sectional study with a relatively small sample size and a very narrow range of fetal gestational age. Echocardiographic analysis of the fetal heart is relatively difficult due to its small size and unfavorable location in the uterine cavity. Second, we did not study diastolic function because the fetal heart rate was close to 150 beats per minute. The rapid heart rate significantly shortened the diastolic period, resulting in poor repeatability of ultrasonic diastolic function measurements. Third, we needed to use multiple acoustic windows to view the right heart precisely from different perspectives; an experienced sonographer was required to complete the parameter measurement. Automatic algorithms for measuring fetal RV function need to be further developed.

## Conclusion

In this study, echocardiography of right ventricular systolic properties was altered in well-controlled GDM fetuses compared with normal gestational age. 2-D SE revealed global and regional differences of strain in RVFW in GDM fetuses. We found heterogeneity in RV dysfunction of GDM fetuses, with no differences among the base, middle and apical segments, this suggest occult diffuse RV myocardial dysfunction. The mechanism of hyperglycemia on fetal cardiac structure and function needs be reached at future, the effect after delivery requires further follow-up studies.

## Data Availability

The datasets used and/or analyzed during the current study are available from the corresponding author on reasonable request. However, all data generated or analyzed during this study are included in this published article.

## Abbreviations

FWT: Free Wall Thickness
FAC: Fractional Area Change
FWLS: Free Wall Longitudinal Strain
GDM: Gestational Diabetes Mellitus
RV: Right Ventricular
2D STE: Two-dimensional Speckle-Tracking Echocardiography
TEI: Tei index
TAPSE: Tricuspid Annulus Plane Systolic Excursion
ICT: Isovolumetric Contraction Time
IRT: Isovolumetric Relaxation Time
ET: Ejection Time
